# Study of the fluorescence and interaction between cyclodextrins and neochlorogenic acid, in comparison with chlorogenic acid

**DOI:** 10.1038/s41598-021-82915-9

**Published:** 2021-02-08

**Authors:** Silvia Navarro-Orcajada, Adrián Matencio, Cristina Vicente-Herrero, Francisco García-Carmona, José Manuel López-Nicolás

**Affiliations:** 1 Departamento de Bioquímica y Biología Molecular-A, Facultad de Biología, Universidad de Murcia, Regional Campus of International Excellence “Campus Mare Nostrum”, 30100 Murcia, Spain; 2 Dipartimento Di Chimica, Università di Torino, via P. Giuria 7, 10125 Turin, Italy

**Keywords:** Biochemistry, Biotechnology

## Abstract

Neochlorogenic acid, a less-studied isomer of chlorogenic acid, has been seen to posses antioxidant, antifungal, anti-inflammatory and anticarcinogenic effects, which makes it an interesting candidate for incorporation in functional foods. However, its poor solubility in water and susceptibility to oxidation make such a task difficult. To overcome that, its encapsulation in cyclodextrins (CDs) is proposed. The fluorescence of neochlorogenic acid in different pH conditions was analyzed, and caffeic acid was proved to be the fluorescent moiety in the molecule. An encapsulation model whereby the ligand poses two potential complexation sites (caffeic and D-(-)-quinic moieties), showed that α-CD and HP-β-CD formed the best inclusion complexes with neochlorogenic acid, followed by M-β-CD, β-CD and γ-CD. Molecular docking with the two best CDs gave better scores for α-CD, despite HP-β-CD providing stabilization through H-bonds. The encapsulation of chlorogenic acid led to a similar CD order and scores, although constants were higher for α-CD, β-CD and M-β-CD, lower for HP-β-CD, and negligible for γ-CD. The protonation state affected these results leading to a different order of CD preference. The solubility and the susceptibility to oxidation of neochlorogenic acid improved after complexation with α-CD and HP-β-CD, while the antioxidant activity of both isomers was maintained.

## Introduction

**Figure 1:**
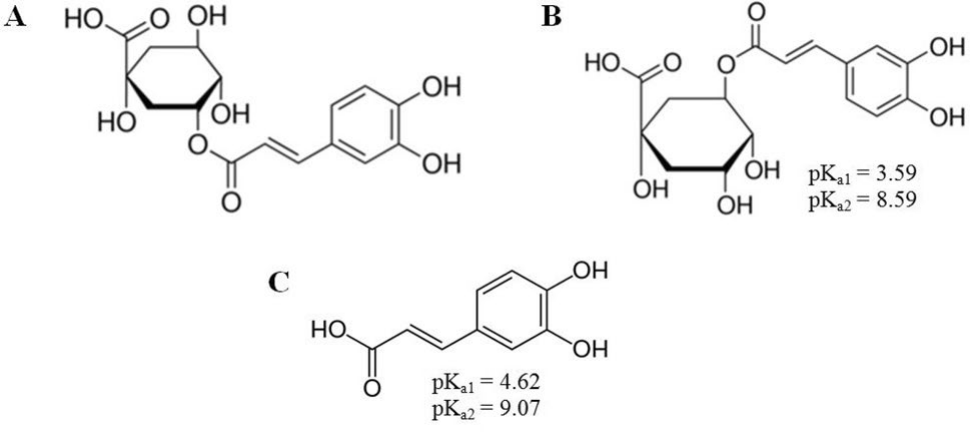
Structure of (**A**) neochlorogenic acid, (**B**) chlorogenic acid and (**C**) caffeic acid; including pKa values determined for the carboxyl group and first phenolic group^45^.

Neochlorogenic acid (3-O-caffeoylquinic acid) is an isomer of chlorogenic acid (5-O-caffeoylquinic acid) formed by ester binding between caffeic acid and D-(-)-quinic acid (Fig. 1). The acid can be found in several foods, such as peaches, prunes, plums, coffee beans, apricots, rosemary leaves and cherries, and was proved to be accumulated throughout thirty days of postharvest drying at room temperature^1–3^.

Although chlorogenic acid is well characterised, its mechanism of action well studied^4,5^ and it is known for its biological properties as an antioxidant, anti-inflammatory, hepatoprotective, antimicrobial, cardioprotective, anticarcinogenic, neuroprotective, anti-obesity, anti-diabetes (among others) agent^6,7^, research about the physicochemical properties and biological effects of its isomer, neochlorogenic acid, is scarce. Despite this, some studies assert that neochlorogenic acid also has antioxidant, antifungal, anti-inflammatory and anticarcinogenic bioactivities^8–13^, and might therefore be an interesting candidate for incorporating in functional foods or nutraceuticals as a bioactive compound.

However, in this respect, the molecule presents problems, including its low solubility in water and susceptibility to oxidation by the enzyme polyphenol oxidase due to its o-diphenol structure^14,15^, making it necessary to find new strategies.

Several studies have demonstrated that the encapsulation of bioactive compounds in cyclodextrins (CDs) is a suitable way to overcome problems of this nature^16,17^. CDs are torus-shaped oligosaccharides made up of α-(1,4) linked glucose units. The most common are natural CDs, α, β and γ-CD, which contain six, seven and eight glucose units, respectively^18^ and are included in the European list of additives approved for alimentary use with the corresponding E-numbers: E-457, E-459 and E-458, respectively. Apart from this, α-CD could be considered the most interesting CD for use in functional foods as it is on the register of EU health claims from the European Food and Safety Authority (EFSA). This claim asserts that the consumption of α-CD as a part of a starch-containing meal reduces the rise in blood glucose levels after that meal^19^. This makes α-CD a suitable ingredient in foodstuffs intended for diabetics.

CDs have a hydrophobic inner cavity due to the orientation of their hydrogen atoms, unlike their mainly hydrophilic outer surface in which the primary and secondary hydroxyl groups are exposed to the solvent, making the whole molecule fairly polar which enables its solubility in aqueous solutions. This fact means that poorly water-soluble compounds and hydrophobic moieties interact non-covalently with the CD inner cavity to form inclusion complexes, which can be more water-soluble than the free form depending on the type of CD used^20^. The use of these encapsulating agents in the food, pharmaceutical and cosmetic industries is rising rapidly due to their ability to increase the bioavailability of different compounds and to protect molecules against the action of external agents^21,22^.

In recent years, our research group has published several works concerning the ability of CDs to encapsulate different molecules of the stilbene family, such as resveratrol, oxyresveratrol, pterostilbene or piceatannol^20,23–26^, lipids^27,28^ and other bioactive compounds^29^. We observed that CDs were able to improve the solubility of phenolic compounds leading to an increase of its activity^24^. Besides that, there are some studies available on the encapsulation of chlorogenic acid by these agents, mainly with β-CD^30–33^, and only a few of them evaluate the impact that this process could have on the activity of the molecule^34,35^. Alvarez-Parilla et al.^30^ proposed a competitive 1:1 model in which a CD molecule could capture chlorogenic acid either by the caffeic acid moiety or the D-(-)-quinic acid moiety, and when the complex is formed another CD cannot be incorporated into the system, excluding a 1:2 model with two molecules of CDs. Shao et al. (2014) and Zhao et al. (2010) demonstrated by H-NMR spectroscopy that cyclodextrins could encapsulate chlorogenic acid by these moieties. Since neochlorogenic acid has the same moieties as chlorogenic acid, this type of complexation could be considered to its isomer. Still, there is no research on the encapsulation of neochlorogenic acid or on its fluorescence properties, which extremely limits the knowledge of this molecule and, therefore, its potential incorporation as an ingredient in foods, cosmetics or drugs.

Indeed, this is the first work to make an exhaustive study of the interaction between neochlorogenic acid and several natural and modified CDs, using fluorimetric techniques and molecular docking to obtain a more accurate physicochemical characterization and finally, analysing the effect that complexation has on the solubility, susceptibility to oxidation and biological activity of this compound. All this accompanied by a comparison with its most investigated isomer, chlorogenic acid, which makes the study more complete and easier to compare with the current literature. Bearing the above in mind, the objectives of this study were:

1. To evaluate the fluorescence of neochlorogenic acid and the reason for the fluorescence.
2. To analyse the encapsulation mechanism of neochlorogenic acid by different types of natural (α-, β- and γ-CD) and modified (HP-β-CD and M-β-CD) CDs.
3. To compare the encapsulation of neochlorogenic acid with the encapsulation of chlorogenic acid.
4. To perform a computational analysis of the encapsulation of neochlorogenic acid with CDs.
5. To analyse the solubility and susceptibility to oxidation of neochlorogenic acid after cyclodextrin complexation.
6. To determine the antioxidant activity of neochlorogenic and chlorogenic acids in the presence and absence of CDs.

## Methods

Neochlorogenic acid was purchased from Baoji Guokang Bio-Technology Co. (Baoji City, China). Chlorogenic acid, caffeic acid and natural CDs (α-, β- and γ-CD) were purchased from Sigma-Aldrich (Madrid, Spain). Modified CDs, 2-hydroxypropyl-β-CD (HP-β-CD, DS = 5) and methyl-β-CD (M-β-CD, DS = 5.4), were purchased from Carbosynth (Berkshire, UK). D-(-)-quinic acid was purchased from Alfa Aesar. 2,2′-diphenyl-1-picrylhydrazyl (DPPH) was purchased from ThermoFisher (Madrid, Spain).

### Studying the fluorescence of neochlorogenic acid

The maximum excitation and emission wavelengths of fluorescence were measured using a Shimazdu RF-6000 Spectrofluorimeter equipped with thermostatically controlled cells, setting both excitation and emission brandwidths at 5 nm.

Fluorescence intensity was measured in a Kontron SFM-25 spectrofluorimeter (Zurich, Switzerland) equipped with thermostatically controlled cells and with a xenon lamp source and quartz cell, which were used to perform all the fluorescence measurements. Excitation and emission bandwidths were both set at 2 nm. The relative fluorescence intensity values were recorded at 25 °C. To avoid inner filter effects, 2 mm quartz cells were used.

For the study of the fluorescence and determination of the encapsulation constants in different pH conditions, 0.1 M acetate, sodium phosphate and tris buffers were used for pH values of 3, 5 and 9, respectively. The concentration of neochlorogenic acid and chlorogenic acid was fixed at 25 µM, both dissolved in water for the encapsulation analysis. The CD concentration was varied between 0 and 11 mM, except for β-CD (between 0 and 8 mM).

### Determination of the encapsulation constant of the inclusion complexes

To determine the stoichiometry of the inclusion complexes formed between neochlorogenic and chlorogenic acids with different cyclodextrins, a competitive model with two potential complexation sites in the guest molecule, one on the caffeic acid moiety and the other one on the D-(-)-quinic acid moiety, was followed. Accordingly, there are two complexation constants for each complexed moiety, K_F1_ and K_F2_. This methodology is similar to that described on Álvarez-Parilla et al.^30^ for chlorogenic acid and Matencio et al.^36^ for a symmetrical molecule, ellagic acid. However, the formation of these two types of complexes is rarely considered in the current literature leading to inaccurate results.

Bearing in mind the above, we obtained two possible chemical balances for both guest moieties:

1 $$ \left( {CD - XA} \right)\underset {{K_{F1}}} \leftrightarrows XA + CD\;\underset {{K_{F2}}} \leftrightarrows \left( {XA - CD} \right)$$

where XA is neochlorogenic acid or chlorogenic acid, (CD-XA) is the inclusion complex on the caffeic acid moiety of neochlorogenic or chlorogenic acid, and (XA-CD) is the inclusion complex on the D-(-)-quinic acid moiety of neochlorogenic or chlorogenic acid. The encapsulation constants of the inclusion complexes (K_F1_ and K_F2_) are given by:

2 $${K}_{F1}=\frac{[CD-XA]}{\left[XA\right] \cdot \left[CD\right]} {K}_{F2}=\frac{[XA-CD]}{\left[XA\right]\cdot \left[CD\right]}$$

where [XA] is the equilibrium concentration of either neochlorogenic acid or chlorogenic acid, [CD] is the equilibrium concentration of CD, and [CD-XA] and [XA-CD] are the equilibrium concentration of the inclusion complexes.

Finally, the model used to analyse the interaction with CDs was:

3 $$(F-{F}_{0})=\frac{{F}_{0} + {(F}_{1}-{F}_{0})\cdot {K}_{F1}\cdot [CD]}{1+({K}_{F2}+{K}_{F1})\cdot [CD]}$$

where F is the experimentally measured fluorescence intensity, F_1_ is the fluorescence intensity of the fully complexed fluorophore, K_F1_ and K_F2_ are the complexation constants for the equilibrium of the caffeic acid or D-(-)-quinic acid moieties, respectively, of neochlorogenic and chlorogenic acids with CDs and [CD] is the initial concentration of CD (assuming that [CD]_equilibrium_ = [CD]_0_ because [CD]_0_ >  > [XA]_0_). The fluorescence in the absence of CDs was normalized to value 1 in the spectrofluorimeter. Equation was run in Sigma-Plot by introducing the normalized values obtained of fluorescence intensity (F) for each cyclodextrin concentration ([CD]), including the normalized basal fluorescence intensity (F_0_ = 1) when [CD] = 0, under following conditions: iterations 10,000,000, step size 100 and tolerance 0.00001. Supplementary Method describes in more detail how to obtain Eq. (3).

The identification of the constants obtained for each moiety of the bioactive compounds (K_F1_ and K_F2_) was figured from the assumption that the molecular moiety that obtains a lower score value in the molecular docking should also have higher encapsulation constants, since both results are related to greater stability of the inclusion complexes.

### Molecular docking

The molecular structures used in this work were built using Avogadro Software^37^ or were obtained from different databases. The α-CD structure was extracted from a crystal from the Protein Data Bank (PDB ID: 2XFY). Neochlorogenic and chlorogenic acids were obtained from the PubChem database (NCBI, USA). HP-β-CD was built by adding hydroxypropyl groups to the β-CD extracted from a crystal from the Protein Data Bank (PDB ID: 1Z0N). The topology of HP-β-CD was obtained using PRODRG with default parameters. Default topology was used for the remaining molecules. Input files for docking were generated using Autodock tools (version 1.5.6) with default parameters and charges. Molecular docking was carried out with Autodock Vina^38^ using default parameters. All CDs were considered as flexible. Graphical representations of the docking results were prepared using PyMOL (Molecular Graphics System, version 1.3, Schrödinger, LLC) with default parameters to display hydrogen bonds.

### Determination of the aqueous solubility

Test tubes containing a saturated concentration of neochlorogenic acid were incubated at increasing concentration of α-CD and HP-β-CD in water using a Thermomixer Comfort system at 1000 rpm and 25 °C in darkness. The neochlorogenic acid concentration was set at 50 mg/mL based on the aqueous solubility of chlorogenic acid (PubChem: 40 mg/mL). After 72 h, they were centrifuged at 5000×*g* for 5 min and the absorbance at λ_max_ 324 nm of the supernatant containing solubilized neochlorogenic acid was measured.

### Determination of enzymatic oxidation of neochlorogenic acid

Enzymatic oxidation was measured after 10 min of reaction with 10 µg/mL neochlorogenic acid at increasing concentration of α-CD and HP-β-CD and 2 µg/mL of polyphenol oxidase at 25 °C. Sodium phosphate 0.1 M pH 6.5 was used as buffer. The absorbance at λ_max_ 324 nm was measured and the percentage of remaining neochlorogenic acid was calculated based on the initial and final values.

### Determination of the antioxidant activity

The measurement of the antioxidant activity of neochlorogenic acid and chlorogenic acid, free and complexed with CDs was made by DPPH method^39^. Solutions of 25 µM neochlorogenic and chlorogenic acids with different concentration of α-CD and HP-β-CD from 0 to 10 mM in sodium phosphate buffer pH 5 were mixed 1:1 with an ethanolic solution of 0.004% (w/v) DPPH. Ethanol was used as a negative control. All samples were incubated 30 min in darkness, and then absorbance was measured at 525 nm in a Bio-Tek Synergy HT plate reader (Winooski). Antioxidant capacity was determined as the percentage of DPPH scavenging activity, given by:

4 $$\% Inhibition=\frac{{Abs}_{control}-{Abs}_{sample}}{{Abs}_{control}} \times 100$$

### Data analysis

All experiments were carried out in triplicate. Regressions were made using Sigma-Plot (version 10.0.0.54), except in the case of the spectra graphics, which were made by the spectrofluorimeter software. A T-test was carried out using Rstudio (version 0.99.878)^40^ with a significance of P < 0.05. Other mathematical operations were carried out using wxMaxima (version 12.04.0).

## Results and discussion

### Studying the fluorescence of neochlorogenic acid effect of pH

First, the maximum excitation and emission wavelengths of fluorescence of neochlorogenic and chlorogenic acids in water were determined, which were 328 and 447 nm, respectively, for neochlorogenic acid, and 327 and 449 nm, respectively, for chlorogenic acid.

Then, the cause of fluorescence was analyzed according to the molecular structure. For this purpose, not only the fluorescence of neochlorogenic and chlorogenic acids was measured individually, but also the fluorescence of the two constituent molecules, caffeic acid and D-(-)-quinic acid. We discovered that in the molecule of interest, the only moiety that was really fluorescent and, therefore, caused neochlorogenic acid to fluoresce, was caffeic acid, since no fluorescence was observed in D-(-)-quinic acid. The fluorescence observed could only have been a result of the polyphenol moiety of this compound^41^.

The next step was to determine whether variation of the pH had any effect on the fluorescence. For this, we measured the fluorescent emission signal of the three acids: neochlorogenic, chlorogenic and caffeic acid, when excited at their maximum wavelength in different pH conditions, from acid to basic (pH 3, 5 and 9). D-(-)-quinic was not selected for this analysis as no fluorescence signal was observed previously.

**Figure 2:**
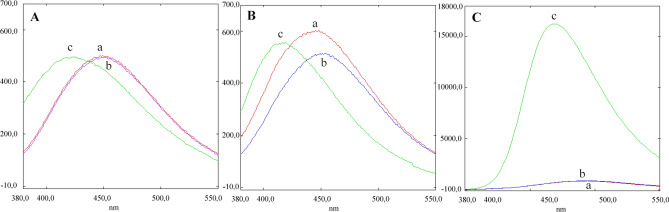
Effect of (**A**) pH 3, (**B**) pH 5 and (**C**) pH 9 on the fluorescence emission signals of (a) neochlorogenic acid, (b) chlorogenic acid and (c) caffeic acid.

Using pH 3 (Fig. 2A) or 5 (Fig. 2B) made almost no difference in the maximum emission wavelengths for neochlorogenic acid and chlorogenic acid; however, the maximum emission wavelength of caffeic acid was 10 nm less at pH 5 compared to pH 3. Conversely, pH 9 (Fig. 2C) induced a clear increase in the maximum emission wavelength of more than 40 nm for every tested compound compared with the values observed at pH 5. Of particular note was the noticeable increase in the fluorescence intensity of caffeic acid at pH 9, which was at least 18 times higher than that of both neochlorogenic and chlorogenic acids at the same pH.

The fluorescent behaviour of caffeic acid at pH 9 in contrast to that of neochlorogenic acid or chlorogenic acid, could be related to a disruption in the internal resonance of caffeic acid due to the disappearance of its first pKa when its carboxyl group is esterified to form neo- or chlorogenic acids^42^. This effect can be observed when the pKa of the hydroxyl group is reached (9.07). To check whether the resonance of caffeic acid was responsible for the increase of fluorescence observed, the fluorescence of dihydrocaffeic acid (which has the same structure as caffeic acid but does not present the internal double bond) was studied. Dihydrocaffeic acid was found to be non-fluorescent, thus confirming that the internal resonance of caffeic acid is essential to maintain its fluorescence^43^, which is affected by the formation of chlorogenic or neochlorogenic acid.

### Determination of the complexation curves for neochlorogenic acid encapsulated in α-CD at pH 5

Bearing in mind the beneficial properties for health of α-CD^19^, we decided to study first the encapsulation of neochlorogenic acid in this cyclodextrin at the intermediate pH 5 and to compare the results with those for chlorogenic acid encapsulated in the same cyclodextrin.

**Figure 3:**
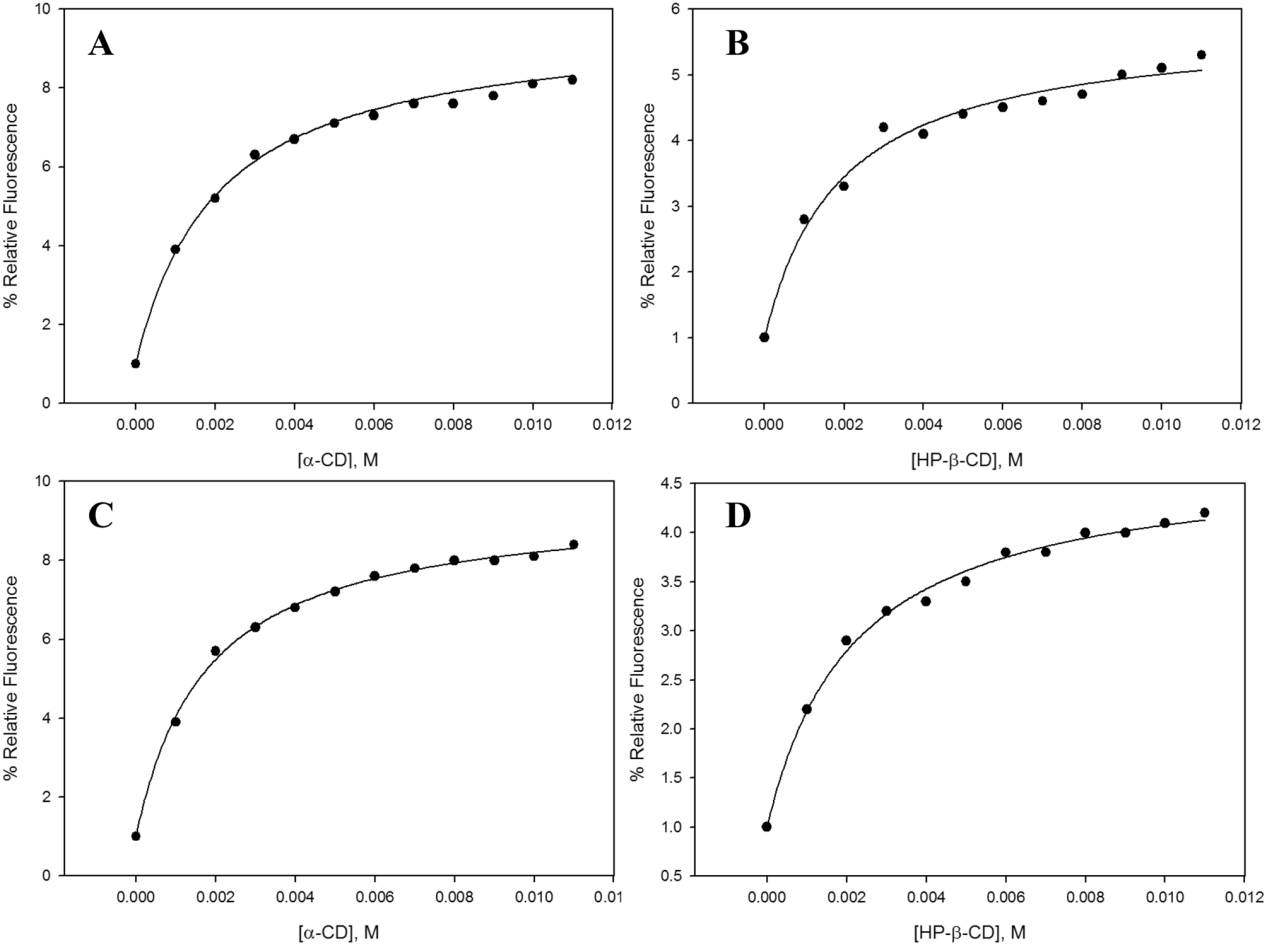
Plotting data, fitted with Eq. (3), of the relative fluorescence signal of neochlorogenic acid vs. (**A**) α-CD and (**B**) HP-β-CD concentration, and the relative fluorescence signal of chlorogenic acid vs. (**C**) α-CD and (**D**) HP-β-CD concentration (25 °C pH 5).

The relative fluorescence of both compounds in the presence of different concentrations of α-CD from 0 to 11 mM was measured (Supplementary Data). Both ligands showed similar complexation curves (Fig. 3A,C), though neochlorogenic acid had lower encapsulation constants (K_F1_ = 457.56 ± 22.88 M^−1^, K_F2_ = 30.64 ± 1.53 M^−1^) than chlorogenic acid (K_F1_ = 530.06 ± 26.50 M^−1^, K_F2_ = 32.63 ± 1.63 M^−1^) (Table 1), indicating that the inclusion complexes between chlorogenic acid and α-CD are more stable than the inclusion complexes between neochlorogenic acid and this type of CD. These results were corroborated using a T-Test with a significance of 0.05.

**Table 1:** Experimental encapsulation constants (K_F1_ and K_F2_) and correlation coefficients (R^2^) arising from Eq. (3) (25 °C pH 3, 5 and 9).

CD type	pH	Neochlorogenic acid			Chlorogenic acid		
		K_F1_ (M^−1^)	K_F2_ (M^−1^)	R^2^	K_F1_ (M^−1^)	K_F2_ (M^−1^)	R^2^
α-CD	3	216.05 ± 10.80	23.22 ± 1.16	0.998	203.66 ± 10.18	20.83 ± 1.04	0.998
	**5**	**457.56 ± 22.88**	**30.64 ± 1.53**	**0.996**	**530.06 ± 26.50**	**32.63 ± 1.63**	**0.997**
	9	490.51 ± 24.53	26.89 ± 1.34	0.989	757.86 ± 37.89	35.68 ± 1.78	0.995
β-CD	3	482.87 ± 24.14	20.53 ± 1.03	0.984	286.59 ± 14.33	15.37 ± 0.77	0.971
	**5**	**159.77 ± 7.99**	**12.71 ± 0.64**	**0.985**	**311.75 ± 15.59**	**14.56 ± 0.73**	**0.975**
	9	70.72 ± 3.54	10.63 ± 0.53	0.984	170.71 ± 8.54	16.38 ± 0.82	0.993
γ-CD	3	29.36 ± 1.47	6.94 ± 0.35	0.982	20.53 ± 1.03	6.46 ± 0.32	0.984
	**5**	**22.42 ± 1.12**	**6.56 ± 0.33**	**0.981**	**0.58 ± 0.03**	**–**	**0.972**
	9	–	–	0.965	25.26 ± 1.26	5.55 ± 0.28	0.984
HP-β-CD	3	538.15 ± 26.91	26.22 ± 1.31	0.976	471.22 ± 23.56	20.43 ± 1.02	0.988
	**5**	**503.74 ± 25.19**	**24.93 ± 1.25**	**0.992**	**439.52 ± 21.98**	**21.20 ± 1.06**	**0.997**
	9	96.96 ± 4.85	14.39 ± 0.72	0.986	163.31 ± 8.17	16.47 ± 0.82	0.980
M-β-CD	3	290.71 ± 14.54	19.24 ± 0.96	0.974	397.49 ± 19.87	19.42 ± 0.97	0.965
	**5**	**314.94 ± 15.75**	**14.31 ± 0.72**	**0.991**	**381.87 ± 19.09**	**20.27 ± 1.01**	**0.987**
	9	127.38 ± 6.37	11.44 ± 0.57	0.974	389.86 ± 19.49	19.41 ± 0.97	0.921

T-test (significance of p < 0.05).

### Selection of the optimum type of cyclodextrin for the encapsulation of neochlorogenic acid at pH 5

After studying the encapsulation of neochlorogenic acid with α-CD, the encapsulation of this bioactive compound using other types of CDs (β-CD, γ-CD, HP-β-CD, M-β-CD) was tested in order to discover which one is the most suitable to encapsulate this molecule.

Table 1 shows the encapsulation constants (K_F1_ and K_F2_) for each complex, as well as the correlation coefficients which were higher than 0.97 in every case. The results showed that both α-CD and HP-β-CD could be considered the best cyclodextrins to encapsulate neochlorogenic acid (K_F1_ values of 457.56 ± 22.88 M^−1^ and 503.74 ± 25.19 M^−1^, and K_F2_ values of 30.64 ± 1.53 M^−1^ and 24.93 ± 1.25 M^−1^, respectively), followed by M-β-CD (K_F1_ = 314.94 ± 15.75 M^−1^ and K_F2_ = 14.31 ± 0.72 M^−1^), β-CD (K_F1_ = 159.77 ± 7.99 M^−1^ and K_F2_ = 12.71 ± 0.64 M^−1^) and finally γ-CD (K_F1_ = 22.42 ± 1.12 M^−1^ and K_F2_ = 6.56 ± 0.33 M^−1^). The complexation curves obtained for the two best CDs (Fig. 3A,B) were adjusted quite well by the mathematical model used, which corroborates the accuracy of the method.

In this way, it was revealed that CDs with a smaller cavity or those modified to provide a greater surface area formed better inclusion complexes than CDs with a larger cavity. These results and the fact that α-CD, as a natural CD, is approved for incorporation in foods and also holds a health claim, unlike the other of CDs used, point to this CD being the optimal one to encapsulate neochlorogenic acid for inclusion in functional foods.

### Isomeric influence on encapsulation with CDs at pH 5

In order to obtain a broader view of how the differences in geometric distribution between neochlorogenic acid and chlorogenic acid affect their encapsulation, the encapsulation constants for the inclusion complexes formed between chlorogenic acid and the previously selected CDs were also determined according to the same methodology used for neochlorogenic acid (Table 1).

The order of the CDs forming the best inclusion complexes with chlorogenic acid remained almost the same as for neochlorogenic acid, except for α-CD, which showed higher constants than HP-β-CD. Still, Fig. 3C,D revealed a very similar behaviour in the complexation curves of chlorogenic acid with respect to those of its isomer (Fig. 3A,B).

Higher constants were observed for chlorogenic acid when encapsulated in α-CD (K_F1_ = 530.06 ± 26.50 M^−1^ and K_F2_ = 32.63 ± 1.63 M^−1^), β-CD (K_F1_ = 311.75 ± 15.59 M^−1^ and K_F2_ = 14.56 ± 0.73 M^−1^) and M-β-CD (K_F1_ = 381.87 ± 19.09 M^−1^ and K_F2_ = 20.27 ± 1.01 M^−1^) than for neochlorogenic acid encapsulated with the same CDs. However, the same behaviour was not observed when the respective acids were encapsulated by other CDs. For example, chlorogenic acid formed complexes with HP-β-CD that provided lower encapsulation constants (K_F1_ = 439.52 ± 21.98 M^−1^ and K_F2_ = 21.20 ± 1.06 M^−1^) than was the case for neochlorogenic acid, while the encapsulation constant of chlorogenic acid with γ-CD was negligible.

Such results confirm that though both neochlorogenic acid and chlorogenic acid have a very similar structure, their slight spatial differences lead to great disparity in their interaction with CDs, intensifying or mitigating the same according to the type of CD. Furthermore, the results of this novel research on the complexation of these isomers with several CDs could lead to obtaining more stable inclusion complexes for application in the food, pharmaceutical or cosmetic industries. Something very useful since most of the literature at the moment is restricted to the use of β-CD to encapsulate chlorogenic acid, ignoring the potential that other CDs, such as α-CD or HP-β-CD, have to complex this type of guest molecules.

### Effect of the protonation state on the encapsulation constants

The previous study was also conducted at a more acidic pH and a basic pH to find out if the protonation state of neochlorogenic acid and its isomer affected the calculated constants (Table 1). Unlike pH 5 shown above, when the carboxyl group was presumably protonated (pH 3), the α-CD constants with both acids were lower, half lower in the case of K_F1_ (K_F1_ = 210.05 ± 10.80 M^−1^ and K_F2_ = 23.22 ± 1.16 M^−1^ for neochlorogenic acid, K_F1_ = 203.66 ± 10.18 M^−1^ and K_F2_ = 20.83 ± 1.04 M^−1^ for chlorogenic acid), the γ-CD constants higher, even twenty times more for chlorogenic acid (K_F1_ = 29.36 ± 1.47 M^−1^ and K_F2_ = 6.94 ± 0.35 M^−1^ for neochlorogenic acid, K_F1_ = 20.53 ± 1.03 M^−1^ and K_F2_ = 6.46 ± 0.32 M^−1^ for chlorogenic acid), the HP-β-CD (K_F1_ = 538.15 ± 26.91 M^−1^ and K_F2_ = 26.22 ± 1.31 M^−1^ for neochlorogenic acid, K_F1_ = 471.22 ± 23.56 M^−1^ and K_F2_ = 20.43 ± 1.02 M^−1^ for chlorogenic acid) and the M-β-CD (K_F1_ = 290.71 ± 14.54 M^−1^ and K_F2_ = 19.24 ± 0.96 M^−1^ for neochlorogenic acid, K_F1_ = 397.49 ± 19.87 M^−1^ and K_F2_ = 19.42 ± 0.97 M^−1^) remained almost stable and the β-CD inclusion complexes gave differences according to the guest molecule. In particular, these β-CD constants with neochlorogenic acid improved, up to three times more for K_F1_ (K_F1_ = 482.87 ± 24.14 M^−1^ and K_F2_ = 20.53 ± 1.03 M^−1^) and were preserved when the guest molecule was chlorogenic acid (K_F1_ = 286.59 ± 14.33 M^−1^ and K_F2_ = 15.37 ± 0.77 M^−1^).

It is highlighted that at pH 3, the best CD to encapsulate neochlorogenic acid was HP-β-CD followed by β-CD, M-β-CD, α-CD and γ-CD, instead of being followed by α-CD, M-β-CD, β-CD and γ-CD as occurred at pH 5. The sequence of the best CDs to form a complex with chlorogenic acid at this pH was similar to that of its isomer, except for β-CD and M-β-CD that permuted positions.

By contrast, this behaviour varied when the hydroxyl group was supposed to be deprotonated (pH 9). In general, at the basic pH (Table 1), the inclusion complexes with neochlorogenic acid revealed lower constants than at pH 5, except for α-CD K_F1_, whose difference was no significant (K_F1_ = 490.51 ± 24.53 M^−1^ and K_F2_ = 26.89 ± 1.34 M^−1^). Some of these reductions were really notorious. For instance, β-CD (K_F1_ = 70.72 ± 3.54 M^−1^ and K_F2_ = 10.63 ± 0.53 M^−1^) and M-β-CD (K_F1_ = 127.38 ± 6.37 M^−1^ and K_F2_ = 11.44 ± 0.57 M^−1^) halved their respective encapsulation constants, HP-β-CD (K_F1_ = 96.96 ± 4.85 M^−1^ and K_F2_ = 14.39 ± 0.72 M^−1^) decreased it 5 times and the constant with γ-CD was now negligible. In the case of complexes with chlorogenic acid at the basic pH the constants with β-CD (K_F1_ = 170.71 ± 8.54 M^−1^ and K_F2_ = 16.38 ± 0.82 M^−1^) and HP-β-CD (K_F1_ = 163.31 ± 8.17 M^−1^ and K_F2_ = 16.47 ± 0.82 M^−1^) were lower than at pH 5. However, α-CD (K_F1_ = 757.86 ± 37.89 M^−1^ and K_F2_ = 35.68 ± 1.78 M^−1^) and γ-CD (K_F1_ = 25.26 ± 1.26 M^−1^ and K_F2_ = 5.55 ± 0.28 M^−1^) provided higher constants while M-β-CD (K_F1_ = 389.86 ± 19.49 M^−1^ and K_F2_ = 19.41 ± 0.97 M^−1^) constants were almost the same.

At pH 9, the order of the best CDs to complex neochlorogenic acid was α-CD followed by M-β-CD, HP-β-CD, β-CD and γ-CD. As occurred before, the CD sequence with its isomer was similar except for the position of β-CD and M-β-CD.

### Molecular docking of the inclusion complexes formed with CDs

**Figure 4:**
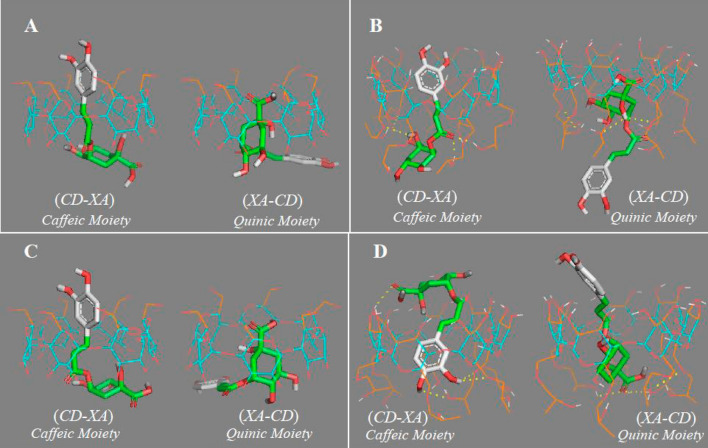
Most likely configurations of the molecular docking of neochlorogenic acid with (**A**) α-CD and (**B**) HP-β-CD, and chlorogenic acid with (**C**) α-CD, and (**D**) HP-β-CD, in the caffeic acid moiety (*CD-XA*) or quinic acid moiety (*XA-CD*). Flexible atoms of CDs are coloured orange and non-flexible atoms blue. Hydrogen bonds are yellow dotted lines.

The molecular modelling of the most likely inclusion complexes formed between neochlorogenic acid and the two best CDs showed a lower score with α-CD than with HP-β-CD (Table 2). Such scores are used to predict the binding force between two docked molecules involving non-covalent interactions; hence, as scores are negative values, the lower the score, the stronger the bond between the molecules. In this case, α-CD provided a better binding force with neochlorogenic acid than did HP-β-CD, although the molecular modelling with this last HP-β-CD pointed to hydrogen bonds (Fig. 4A,B), one of the most important interactions in inclusion complexes. This slight difference with respect to the fluorescent study could be due to a variation in the position of the hydroxypropyl groups in the host molecule.

**Table 2:** Results for molecular docking with the two best CDs classified by the encapsulated moiety of the ligand.

CD type	Neochlorogenic acid		Chlorogenic acid	
	Score *(CD-XA) CaffeicMoiety*	Score *(XA-CD) QuinicMoiety*	Score *(CD-XA) CaffeicMoiety*	Score *(XA-CD) QuinicMoiety*
α-CD	− 7.4	− 6.1	− 7.0	− 6.0
HP-β-CD	− 6.7	− 6.4	− 6.3	− 5.8

Moreover, molecular docking of chlorogenic acid with both CDs (Fig. 4C,D) demonstrated that α-CD formed a stronger bond than HP-β-CD. In a comparison of both acids, the scores for neochlorogenic acid were slightly lower than the scores for chlorogenic acid, meaning that the inclusion complexes with neochlorogenic acid seem to be more stable than those with its isomer, since the lower the score, the greater the binding force (Table 2).

It seems the smaller cavity of the natural CD helps to increase the binding force with the guest molecule, slightly more than the modified CD with its greater surface area, which was stabilised by hydrogen bonding.

The inclusion complexes with the caffeic acid moiety of both neochlorogenic and chlorogenic acids (Fig. 4) had better scores than those formed in the D-(-)-quinic moiety (Table 2); hence, the higher encapsulation constant values obtained in Eq. (3) were assumed to be K_F1_, which refers to encapsulation in the caffeic acid moiety.

### Solubility of neochlorogenic acid in water after encapsulation in CDs

**Figure 5:**
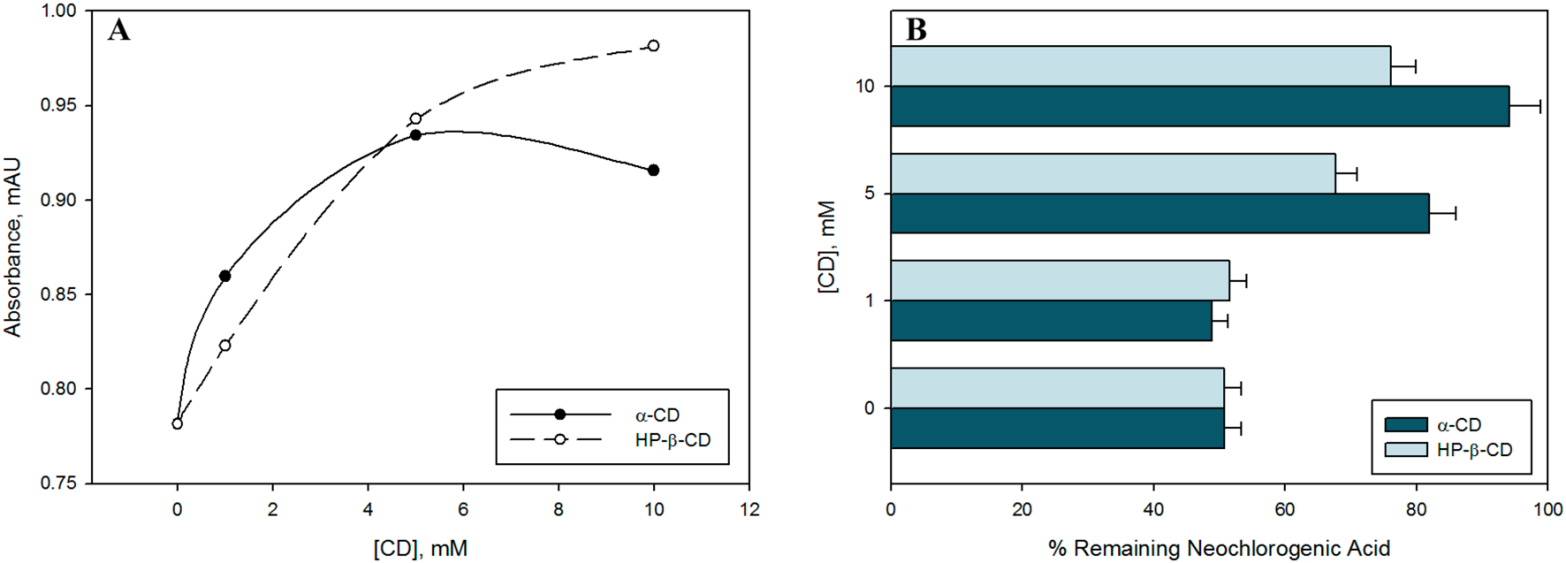
(**A**) Aqueous solubility and (**B**) enzymatic oxidation with polyphenol oxidase of neochlorogenic acid in the presence of α-CD and HP-β-CD (λ_max_ = 324 nm). T-test (significance of p < 0.05).

After three days of incubation, the aqueous solubility of neochlorogenic acid in the presence of α-CD and HP-β-CD improved significantly, giving respectively, a 17% and 26% more of soluble compound with the higher concentration in comparison with the tubes with free neochlorogenic acid (Fig. 5A). The differences between the control without CD and the intermediate and high concentration of both CDs were significant. Overall, HP-β-CD gave the best results, despite α-CD being more effective at the lowest concentration analysed.

### Enzymatic oxidation of neochlorogenic acid free and complexed with CDs

Cyclodextrins were proved to successfully protect neochlorogenic acid against the oxidation by the browning enzyme, polyphenol oxidase. After ten minutes reaction, almost the 50% of the free bioactive compound is lost. Meanwhile, the complexation with the higher concentration of HP-β-CD reduced this percentage to 24%, and even less than 6% with α-CD (Fig. 5B). The differences between the control without CD and the treatments with 5 mM and 10 mM of CD were significant.

In this case, α-CD showed better outcomes than HP-β-CD using an intermediate or high concentration of cyclodextrin. It seems that the inclusion complexes formed with a smaller cavity of the host could provide a better barrier to prevent the bioactive compound enter the active site of the enzyme. In contrast, the lowest concentration gave no significant variation in the remaining amount of neochlorogenic acid in relation to the control without cyclodextrins (Fig. 5B). Therefore, this effect is strongly dependent of the concentration of the encapsulating agent. Billaud et al.^44^ also reported this concentration dependence on enzymatic oxidation of inclusion complexes of chlorogenic acid with β-CD.

### Antioxidant activity of neochlorogenic and chlorogenic acids in the presence and absence of CDs

**Figure 6:**
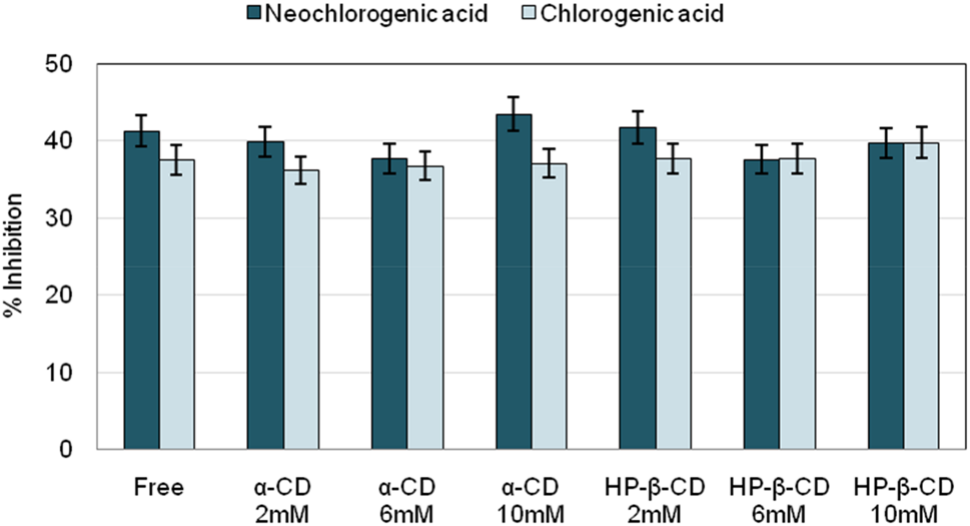
Antioxidant activity of neochlorogenic acid and chlorogenic acid, in free forms and encapsulated in α-CD and HP-β-CD. Ethanol was used as a negative control. T-test (significance of p < 0.05).

The measurement of the percentage of DPPH scavenging activity of these bioactive compounds revealed that the formation of the inclusion complexes with either α-CD or HP-β-CD was able to maintain the original antioxidant activity of both acids, without significant differences among the CD concentrations tested (Fig. 6).

These results are in agreement with the study of Zhao et al.^35^, which analysed the inclusion complexes of chlorogenic acid with β-CD by the same method and observed an apparent dose-dependent effect. Other authors^34^ reported an increase in the antioxidant activity of chlorogenic acid encapsulated in β-CD by FRAP method. However, the concentration of the guest molecule in this last study was ten times higher than the concentration in our research. There are no previous studies to contrast the antioxidant activity of inclusion complexes with neochlorogenic acid.

When comparing the isomers, it highlights that neochlorogenic acid, with or without CDs, had slightly higher antioxidant activity than chlorogenic acid. Despite not being significant at the concentrations tested, these results are of great interest for future investigations on this less-studied isomer of chlorogenic acid.

## Conclusions

To summarise, this study presents a physicochemical and computational study of the encapsulation of neochlorogenic acid, a bioactive isomer of chlorogenic acid. The fluorescence of this compound in different pH conditions was characterized, as well as the structural background of its fluorescence, which showed that caffeic acid was the main fluorescent moiety in the molecule.

At pH 5, the encapsulation results showed that HP-β-CD and α-CD formed the best inclusion complexes with neochlorogenic acid, followed by M-β-CD, β-CD and finally γ-CD. Molecular docking with the two best CDs provided better scores for α-CD, although HP-β-CD provided stabilisation through hydrogen bonds. As α-CD is authorized for inclusion in food products and also holds a health claim, this CD should be considered in preference to HP-β-CD for encapsulating neochlorogenic acid in order to design functional foods or nutraceuticals.

A comparison with the encapsulation of chlorogenic acid with the same CDs pointed to a similar order of constants and docking scores, although the encapsulation constants were higher for α-CD, β-CD and M-β-CD, lower for HP-β-CD, and negligible for γ-CD. These findings confirm that, while neochlorogenic and chlorogenic acid have a very similar structures, their slight spatial differences leads to a wide great disparity in their interaction with CDs.

The protonation state was revealed to affect encapsulation constants, leading to a different order of preference of CD to form inclusion complexes. Compared with neochlorogenic acid at pH 5, the positions of α-CD and β-CD were permuted at a more acidic pH, while at basic pH α-CD, M-β-CD and HP-β-CD gave the more stable inclusion complexes.

The solubility of neochlorogenic acid in water was shown to improve in the presence of CDs, with HP-β-CD being more suitable than α-CD for this purpose. In addition, this complexation proved to be capable of protecting neochlorogenic acid against enzymatic oxidation by polyphenol oxidase, maintaining up to 94% of the bioactive compound compared to 50% when it is in free form. In this sense, α-CD was able to preserve the molecule better than HP-β-CD.

Finally, the study of the antioxidant activity of both acids in the presence and absence of α-CD and HP-β-CD, revealed that this type of complexation was able to maintain the original activity of neochlorogenic acid and chlorogenic acid, independently of the CD concentration tested. Neochlorogenic acid (free or encapsulated) showed slightly better activity than its isomer, despite not being a significant difference.

Overall, the results of this novel research on the complexation of these isomers with several CDs could lead to obtaining more stable inclusion complexes for application in the food, pharmaceutical or cosmetic industries.

## Supplementary information

Supplementary information 1.

Supplementary information 2.
