# Supplementary material for: Study of the fluorescence and interaction between cyclodextrins and neochlorogenic acid, in comparison with chlorogenic acid
Source: Sci Rep. 2021 Feb 8;11:3275. doi: 10.1038/s41598-021-82915-9 (PMC7870928; doi:10.1038/s41598-021-82915-9)
Supplement: Supplementary file 1 — Supplementary information 1. [file 41598_2021_82915_MOESM1_ESM.pdf]

STUDY OF THE FLUORESCENCE AND INTERACTION BETWEEN CYCLODEXTRINS AND NEOCHLOROGENIC ACID, IN COMPARISON WITH CHLOROGENIC ACID

Silvia Navarro-Orcajada [1], Adrián Matencio [2], Cristina Vicente-Herrero [1], Francisco García-Carmona [1] and José Manuel López-Nicolás [1]*

[1] Departamento de Bioquímica y Biología Molecular-A, Facultad de Biología, Universidad de Murcia - Regional Campus of International Excellence "Campus Mare Nostrum, E-30100 Murcia, Spain

[2] Dipartimento Di Chemica, Università di Torino, via P. Giuria 7, 10125 Torino, Italy

* Corresponding author: Tel: 34 868 884777 Fax: 34 868 364147

E-mail: josemln@um.es

Supplementary Data. Raw data for fluorescence intensity of neochlorogenic acid (N) and chlorogenic acid (C) at increasing cyclodextrin concentration.

**pH**	**Guest**	**Host**	**[CD], mM**											
			**0**	**1**	**2**	**3**	**4**	**5**	**6**	**7**	**8**	**9**	**10**	**11**
5	N	α-CD	1.0	3.9	5.2	6.3	6.7	7.1	7.3	7.6	7.6	7.8	8.1	8.2
		β-CD	1.0	1.5	1.9	2.0	2.2	2.3	2.5	2.7	2.8	-	-	-
		γ-CD	1.0	1.1	1.3	1.3	1.6	1.8	1.8	1.9	1.9	1.9	2.0	2.1
		HP-β-CD	1.0	2.8	3.3	4.2	4.2	4.4	4.5	4.6	4.7	5.0	5.1	5.3
		M-β-CD	1.0	1.3	1.5	1.8	1.9	1.9	1.9	2.0	2.0	2.1	2.1	2.1
	C	α-CD	1.0	3.9	5.7	6.3	6.8	7.2	7.6	7.8	8.0	8.0	8.1	8.4
		β-CD	1.0	1.3	1.8	1.9	1.9	1.9	2.0	2.2	2.2	-	-	-
		γ-CD	1.0	1.0	1.1	1.1	1.2	1.2	1.2	1.3	1.4	1.5	1.6	1.6
		HP-β-CD	1.0	2.2	2.9	3.2	3.3	3.5	3.8	3.8	4.0	4.0	4.1	4.2
		M-β-CD	1.0	2.1	2.5	3.0	3.1	3.2	3.3	3.3	3.5	3.7	3.7	3.8
3	N	α-CD	1.0	3.0	4.4	5.7	6.3	6.9	7.2	7.7	7.9	8.4	8.7	9.0
		β-CD	1.0	1.7	2.1	2.5	2.6	2.7	2.8	2.9	2.9	-	-	-
		γ-CD	1.0	1.3	1.3	1.4	1.6	1.7	1.9	2.0	2.3	2.3	2.4	2.5
		HP-β-CD	1.0	2.9	3.4	3.8	4.0	4.0	4.2	4.3	4.5	4.6	4.6	4.7
		M-β-CD	1.0	2.3	2.7	2.9	3.3	3.4	3.5	3.8	3.8	3.9	4.0	4.0
	C	α-CD	1.0	2.6	3.7	4.3	5.3	5.7	6.1	6.3	6.6	6.9	7.1	7.2
		β-CD	1.0	1.5	1.7	1.9	2.0	2.3	2.4	2.4	2.4	-	-	-
		γ-CD	1.0	1.3	1.4	1.4	1.5	1.6	1.8	1.9	2.0	2.2	2.3	2.4
		HP-β-CD	1.0	1.8	2.4	2.6	2.8	2.9	2.9	2.9	3.0	3.0	3.0	3.2
		M-β-CD	1.0	1.9	2.4	2.7	2.7	2.7	2.8	3.0	3.2	3.3	3.3	3.4
9	N	α-CD	1.0	3.3	3.9	4.3	4.6	4.8	5.2	5.3	5.4	5.6	5.7	5.8
		β-CD	1.0	1.4	1.7	1.9	2.3	2.4	2.6	2.7	2.8	-	-	-
		γ-CD	1.0	1.0	1.0	1.2	1.3	1.5	1.8	1.9	1.9	1.9	2.1	2.2
		HP-β-CD	1.0	2.0	2.8	3.2	3.4	3.9	4.0	4.3	4.5	4.6	5.0	5.4
		M-β-CD	1.0	1.3	1.8	1.9	1.9	2.1	2.2	2.3	2.3	2.4	2.5	2.7
	C	α-CD	1.0	3.9	5.3	5.7	5.9	6.2	6.4	6.5	6.6	6.8	7.0	7.0
		β-CD	1.0	1.4	1.8	2.2	2.3	2.5	2.6	2.7	2.7	-	-	-
		γ-CD	1.0	1.1	1.4	1.6	1.6	1.6	1.7	1.8	2.0	2.1	2.2	2.4
		HP-β-CD	1.0	2.2	2.7	3.1	3.4	3.6	3.8	4.0	4.1	4.3	4.5	4.8
		M-β-CD	1.0	1.6	1.7	1.7	1.7	1.7	1.8	1.8	1.9	2.0	2.0	2.1
