# Supplementary material for: Study of the fluorescence and interaction between cyclodextrins and neochlorogenic acid, in comparison with chlorogenic acid
Source: Sci Rep. 2021 Feb 8;11:3275. doi: 10.1038/s41598-021-82915-9 (PMC7870928; doi:10.1038/s41598-021-82915-9)
Supplement: Supplementary file 2 — Supplementary information 2. [file 41598_2021_82915_MOESM2_ESM.pdf]

STUDY OF THE FLUORESCENCE AND INTERACTION BETWEEN CYCLODEXTRINS AND NEOCHLOROGENIC ACID, IN COMPARISON WITH CHLOROGENIC ACID

Silvia Navarro-Orcajada [1], Adrián Matencio [2], Cristina Vicente-Herrero [1], Francisco García-Carmona [1] and José Manuel López-Nicolás [1]*

[1] Departamento de Bioquímica y Biología Molecular-A, Facultad de Biología, Universidad de Murcia - Regional Campus of International Excellence "Campus Mare Nostrum, E-30100 Murcia, Spain

[2] Dipartimento Di Chemica, Università di Torino, via P. Giuria 7, 10125 Torino, Italy

* Corresponding author: Tel: 34 868 884777 Fax: 34 868 364147

E-mail: josemln@um.es

Supplementary Method. Competitive 1:1 model for the encapsulation of neochlorogenic acid or chlorogenic acid in cyclodextrins.

In this method, two possible chemical balances for both guest moieties (caffeic acid and D-(-)-quinic acid) are proposed:

$$(CD-XA) \underset{K_{F1}}{\rightleftarrows} XA+CD \underset{K_{F2}}{\leftrightarrows} (XA-CD) \tag{1}$$

where XA is a molecule of neochlorogenic acid or chlorogenic acid, CD is a molecule of cyclodextrin, (CD-XA) is the inclusion complex on the caffeic acid moiety of neochlorogenic or chlorogenic acid, and (XA-CD) is the inclusion complex on the D-(-)-quinic acid moiety of neochlorogenic or chlorogenic acid.

Hence, the encapsulation constants of the inclusion complexes (K_{F1} and K_{F2}) are given by:

$$K_{F1}=\frac{[CD-XA]}{[XA]\cdot[CD]} \qquad K_{F2}=\frac{[XA-CD]}{[XA]\cdot[CD]} \tag{2}$$

where [XA] is the equilibrium concentration of either neochlorogenic acid or chlorogenic acid, [CD] is the equilibrium concentration of CD, and [CD-XA] and [XA-CD] are the equilibrium concentration of the inclusion complexes.

Then, we get the following molar ratios for the molecules obtained in the previous equilibrium:

$$x(XA)=\frac{[XA]}{[XA]+[CD-XA]+[XA-CD]} \tag{3}$$

$$x(CD-XA)=\frac{[CD-XA]}{[XA]+[CD-XA]+[XA-CD]} \tag{4}$$

$$x(XA-CD)=\frac{[XA-CD]}{[XA]+[CD-XA]+[XA-CD]} \tag{5}$$

Considering equations (2) for both encapsulation constants (K_{F1} and K_{F2}), they change to:

$$x(XA) = \frac{[XA]}{[XA]+K_{F1}\cdot[XA]\cdot[CD]+K_{F2}\cdot[XA]\cdot[CD]} = \frac{1}{1+K_{F1}\cdot[CD]+K_{F2}\cdot[CD]} \quad (6)$$

$$x(CD-XA) = \frac{K_{F1}\cdot[XA]\cdot[CD]}{[XA]+K_{F1}\cdot[XA]\cdot[CD]+K_{F2}\cdot[XA]\cdot[CD]} = \frac{K_{F1}\cdot[CD]}{1+K_{F1}\cdot[CD]+K_{F2}\cdot[CD]} \quad (7)$$

$$x(XA-CD) = \frac{K_{F2}\cdot[XA]\cdot[CD]}{[XA]+K_{F1}\cdot[XA]\cdot[CD]+K_{F2}\cdot[XA]\cdot[CD]} = \frac{K_{F2}\cdot[CD]}{1+K_{F1}\cdot[CD]+K_{F2}\cdot[CD]} \quad (8)$$

As caffeic acid is the fluorescent moiety of the guest molecule, when the concentration of CD increases, the experimental measured change in the fluorescence intensity (F-F_0) can be obtained as a summation of the fluorescence intensity in free form and fully complexed by this moiety:

$$(F-F_0) = F_0 \cdot x(XA) + (F_1 - F_0) \cdot x(CD-XA) \quad (9)$$

where F is the experimentally measured fluorescence intensity, F_0 is the fluorescence in the absence of CDs (neochlorogenic or chlorogenic acid in free form) and F_1 is the fluorescence intensity of the fully complexed fluorophore, the caffeic acid moiety.

Considering equations (6) and (7), the previous equation (9) would change to:

$$(F-F_0) = F_0 \cdot \frac{1}{1+K_{F1}\cdot[CD]+K_{F2}\cdot[CD]} + (F_1 - F_0) \cdot \frac{K_{F1}\cdot[CD]}{1+K_{F1}\cdot[CD]+K_{F2}\cdot[CD]} \quad (10)$$

$$(F-F_0) = \frac{F_0+(F_1-F_0)\cdot K_{F1}\cdot[CD]}{1+K_{F1}\cdot[CD]+K_{F2}\cdot[CD]} \quad (11)$$

As neochlorogenic and chlorogenic acids have basal fluorescence intensity in the absence of cyclodextrins, F_0 was normalized to a value of 1 in the spectrofluorimeter.
